# Zona Pellucida Thickness, Reproductive Aging, and Oocyte Competence in Women with Diminished Ovarian Reserve Undergoing ICSI

**DOI:** 10.3390/biomedicines14081772

**Published:** 2026-08-06

**Authors:** Elif Yelda Bayar, Ömer Faruk Öz, Hatice Şiyzen Çoban, Özgür Uzun, Belgin Devranoğlu

**Affiliations:** 1 Department of Obstetrics and Gynecology, Zeynep Kamil Women and Children’s Diseases Training and Research Hospital, University of Health Sciences, Zeynep Kamil Mah., Dr. Burhanettin Üstünel Cad. No. 6E, Üsküdar, 34668 Istanbul, Türkiye; 2 Department of Obstetrics and Gynecology, Faculty of Medicine, Akdeniz University, Dumlupınar Boulevard, Campus, 07070 Antalya, Türkiye

**Keywords:** zona pellucida, reproductive aging, oocyte competence, diminished ovarian reserve, intracytoplasmic sperm injection, assisted reproduction

## Abstract

**Background/Objectives:** The zona pellucida (ZP) is an extracellular matrix surrounding the oocyte. It is unclear whether mean ZP thickness is associated with reproductive age or developmental competence in women with diminished ovarian reserve (DOR). We examined pre-ICSI ZP thickness in relation to age, ovarian response, and early reproductive outcomes. **Methods:** This prospective observational study included 255 women aged 22–46 years with clinically diagnosed DOR who underwent intracytoplasmic sperm injection. ZP thickness was measured at three equidistant points before injection, independently verified by two observers, and summarized as the patient-level mean across the available metaphase II oocytes. Women aged <35 and ≥35 years were compared. The association between ZP thickness and chronological age was also evaluated using Spearman correlation. A transfer-adjusted logistic regression model evaluated beta-hCG positivity using age group, mean ZP thickness, laser-assisted hatching, day-5 transfer, and metaphase II oocyte count. **Results:** Median patient-level mean ZP thickness was similar in women aged <35 and ≥35 years (36.99 [34.90–40.61] vs. 36.91 [34.04–40.87] µm; *p* = 0.875). Mean ZP thickness was not correlated with chronological age (Spearman’s ρ = −0.059; *p* = 0.347). Younger women had higher AMH levels, antral follicle counts, retrieved oocyte counts, and metaphase II oocyte counts. Beta-hCG positivity was also higher in younger women (31.8% vs. 14.6%; *p* = 0.006). In the adjusted model, ZP thickness was not independently associated with beta-hCG positivity (OR 1.03 per µm, 95% CI 0.95–1.11; *p* = 0.487), whereas age ≥ 35 years was associated with lower odds of beta-hCG positivity. **Conclusions:** In this DOR cohort, mean pre-ICSI ZP thickness was not associated with chronological age, either as a continuous variable or after categorization at 35 years. It was also not independently associated with beta-hCG positivity. These findings do not exclude age-related changes in other structural, mechanical, or dynamic properties of the ZP that were not measured in this study.

## 1. Introduction

Diminished ovarian reserve (DOR) is characterized by a reduced number of available follicles and a lower expected response to ovarian stimulation. However, ovarian reserve and oocyte competence are not equivalent. Ovarian reserve tests mainly estimate the expected quantitative response, whereas chronological age remains an important determinant of reproductive potential [1,2,3,4]. The causes and clinical consequences of DOR vary among patients and assisted reproduction settings [5,6]. In addition, oocyte development is influenced by the follicular fluid and the cumulus–oocyte microenvironment [7]. Therefore, structural features of the oocyte may provide information that is not captured by ovarian reserve markers alone.

The zona pellucida (ZP) is an extracellular glycoprotein matrix surrounding the oocyte and preimplantation embryo [8,9,10,11]. It supports oocyte growth and communication between the oocyte and granulosa cells. It also participates in sperm recognition, prevention of polyspermy, protection of the early embryo, and hatching before implantation [8,9,10,11]. Ultrastructural studies have demonstrated its complex three-dimensional fibrillar organization [12,13]. Experimental and genetic studies further suggest that abnormal ZP protein assembly or cross-linking can impair folliculogenesis, fertilization, and early development [14,15]. These findings support the view that the ZP is an active component of the oocyte phenotype rather than a passive shell.

Whether ZP thickness itself captures this biology remains uncertain. Earlier studies reported associations between ZP thickness or thickness variation and embryo quality, implantation, or clinical pregnancy, whereas other studies found modest or inconsistent relationships with female age and reproductive outcomes [16,17,18,19,20,21]. Direct comparison is complicated by measurement at different developmental stages, including metaphase II oocytes, zygotes, cleavage-stage embryos, and blastocysts, and by the use of average thickness, regional variation, density, birefringence, or qualitative appearance as non-equivalent exposures [16,17,18,19,20,21,22].

Qualitative ZP phenotypes, including dark, heterogeneous, thickened, indented, and agar-like zonae, have been associated with impaired maturity, fertilization, embryo development, or pregnancy in selected cohorts [23,24,25,26,27]. At the same time, standardized embryo assessment and image-based morphometric work caution against overinterpreting a single static descriptor outside a broader embryological context [28,29]. This distinction is especially important for simple mean thickness, which cannot directly represent matrix composition, porosity, elasticity, asymmetry, or dynamic remodeling.

Intracytoplasmic sperm injection (ICSI) offers a useful biological context for testing this question because sperm binding and penetration through the ZP are bypassed. Assisted hatching is also directed at the ZP, yet systematic reviews and randomized evidence do not support indiscriminate use across all IVF/ICSI patients [30,31,32]. We therefore asked whether patient-level mean pre-ICSI ZP thickness represents an age-related oocyte phenotype or a biological correlate of oocyte competence in women with DOR. Specifically, we evaluated its relationship with reproductive age, ovarian response, fertilization, and early reproductive outcomes, while accounting for transfer timing and laser-assisted hatching in adjusted analyses.

## 2. Materials and Methods

### 2.1. Study Design and Setting

This prospective observational study was conducted at the IVF/ICSI unit of the University of Health Sciences, Zeynep Kamil Women and Children’s Diseases Training and Research Hospital, Istanbul, Türkiye, between 2024 and 2025. The study examined pre-ICSI ZP thickness in relation to reproductive age, ovarian response, and early reproductive outcomes in women clinically diagnosed with DOR.

### 2.2. Ethics

The study was approved by the Zeynep Kamil Hospital Non-Interventional Research Ethics Committee (approval number: 26; approval date: 10 July 2024). All participants provided written informed consent before inclusion. The study was conducted in accordance with the Declaration of Helsinki, and data were analyzed in anonymized form.

### 2.3. Participants and Ovarian Reserve Phenotype

Eligible participants were women aged 21–46 years with primary or secondary infertility attributed to clinically diminished ovarian reserve, available clinical and laboratory data sufficient for eligibility assessment, and planned ICSI. The final analytic cohort comprised women aged 22–46 years with analyzable ZP measurements. DOR was diagnosed in routine clinical practice using anti-Müllerian hormone (AMH) and antral follicle count (AFC) in the context of the infertility evaluation. A single prespecified AMH or AFC cutoff was not used as an enrollment rule. Accordingly, the cohort should be interpreted as a clinically characterized DOR population rather than a Bologna-defined poor ovarian response or POSEIDON low-prognosis group [33,34]. To make the ovarian reserve phenotype transparent, the observed AMH and AFC distributions and commonly used low-AMH thresholds are reported descriptively.

Exclusion criteria were previous abdominal surgery for an inflammatory condition, relevant chronic systemic disease, chronic medication use, smoking, alcohol or illicit substance use, psychiatric disease that could materially affect study participation, age outside the prespecified range, polycystic ovary syndrome, male factor infertility, and incomplete ZP measurement data. Participants were categorized by reproductive age as <35 years and ≥35 years.

### 2.4. Ovarian Stimulation, Oocyte Retrieval, and ICSI

Controlled ovarian stimulation was performed using subcutaneous follitropin alfa (GONAL-f; Merck, Darmstadt, Germany). The initial daily dose ranged from 150 to 225 IU and was individualized according to the participant’s age, body mass index, serum anti-Müllerian hormone level, and antral follicle count. The gonadotropin dose was subsequently adjusted according to the ovarian response during stimulation monitoring. A flexible gonadotropin-releasing hormone antagonist protocol was used. Subcutaneous cetrorelix acetate (Cetrotide; Merck) at a dose of 0.25 mg/day was initiated when the leading follicle reached a diameter of 13–14 mm and was continued until the day of triggering. Final oocyte maturation was induced with a single subcutaneous dose of 250 µg choriogonadotropin alfa (Ovitrelle; Merck) when at least two follicles reached a diameter of 17–18 mm. Transvaginal oocyte retrieval was performed 35–36 h after the trigger injection. After denudation with hyaluronidase, only metaphase II (MII) oocytes with a visible polar body were selected for ICSI. Fertilization was assessed 16–18 h after injection by the presence of two pronuclei (2PN). Luteal-phase support was initiated on the day after oocyte retrieval using vaginal progesterone gel (Crinone 8%, 90 mg) once or twice daily according to the treating physician’s clinical judgment and was continued without interruption until serum beta-hCG testing, performed approximately 10–12 days after embryo transfer.

### 2.5. Zona Pellucida Measurement and Observer Verification

ZP thickness was measured immediately before ICSI using an Olympus IX53 inverted microscope (Olympus Corporation, Tokyo, Japan) with digital imaging and DPE-BSW software (version 3.3.1.1.198). For each MII oocyte, the distance between the inner and outer ZP boundaries was measured at three approximately equidistant positions around the oocyte circumference (approximately 0°, 120°, and 240°). The three measurements were averaged to obtain the mean ZP thickness for that oocyte.

Each measurement was independently verified by two observers. When the between-observer difference exceeded 5%, the oocyte was remeasured, and the discrepancy was resolved before the value was entered into the study dataset. For patient-level analyses, the primary exposure was the mean ZP thickness across all measured MII oocytes from the participant. This strategy avoided treating multiple oocytes from the same woman as statistically independent observations. Observer-specific raw measurements were not retained in the analytic dataset; therefore, a formal intraclass correlation coefficient could not be reconstructed retrospectively.

### 2.6. Embryo Culture, Transfer Context, and Outcomes

After ICSI, embryos were cultured in sequential G-1/G-2 media (Vitrolife, Gothenburg, Sweden) under standard incubator conditions. Embryo development and quality were assessed using the Istanbul consensus framework [29]. Transfer day was recorded as day 2, 3, 4, or 5 after fertilization. For adjusted analyses, transfer timing was dichotomized as day 5 versus days 2–4. Because embryo developmental stage was not separately encoded in the source dataset, this variable was treated as a timing proxy rather than a direct morphological stage classification. Laser-assisted hatching (LAH) and embryo transfer were performed according to clinical and embryological indications. The source dataset did not contain a validated variable distinguishing fresh from frozen-thawed transfer; transfer-cycle type could therefore not be included in adjusted models.

Embryological outcomes included the numbers of retrieved oocytes, MII oocytes, and 2PN embryos, together with maturation and fertilization rates. Early reproductive outcomes included serum beta-hCG positivity 12 days after embryo transfer, gestational sac visualization by transvaginal ultrasonography, and first-trimester miscarriage up to 14 gestational weeks.

### 2.7. Statistical Analysis, Missing Data, and Model Design

Continuous variables were summarized as median and interquartile range or mean and standard deviation, as appropriate. Categorical variables were summarized as counts and percentages. Between-group comparisons used the Mann–Whitney U test for continuous variables and the chi-square or Fisher’s exact test for categorical variables. Spearman correlation was used to examine associations between patient-level mean ZP thickness and continuous clinical variables. All tests were two-sided, and *p* < 0.05 was considered statistically significant.

For beta-hCG positivity, the prespecified biological exposure of interest was patient-level mean ZP thickness. The multivariable model included age group, mean ZP thickness, LAH, transfer timing (day 5 vs. days 2–4), and MII oocyte count. AMH and MII oocyte count were not entered simultaneously because both represent ovarian reserve or response constructs and were moderately correlated in the cohort (Spearman rho = 0.379; *p* < 0.001). A sensitivity model substituted AMH for MII oocyte count. Model complexity was deliberately restricted because only 41 beta-hCG-positive events were available.

Outcome denominators were handled by available-case analysis. Of 188 women who underwent embryo transfer, 181 had a recorded beta-hCG result. Among 41 beta-hCG-positive pregnancies, 40 had a recorded gestational sac assessment. Among 35 pregnancies with a visualized gestational sac, 30 had recorded first-trimester miscarriage follow-up. No outcome values were imputed. Reasons for missing follow-up were not systematically coded; therefore, the missing data mechanism could not be assumed to be missing at random. Reporting was aligned with the STROBE recommendations for observational studies [35].

### 2.8. Sample Size Sensitivity

No a priori sample size calculation was documented in the original study protocol. The analysis used the full recruited cohort with analyzable patient-level ZP measurements (*n* = 255). As a sensitivity assessment, the achieved age group sizes of 117 and 138 provide 80% power to detect a standardized between-group difference of approximately Cohen’s d = 0.35 under a two-sided alpha = 0.05 comparison framework. For the beta-hCG models, the limited number of positive events constrained model complexity; adjusted effect estimates were therefore interpreted with their 95% confidence intervals rather than significance alone.

## 3. Results

### 3.1. Cohort and Ovarian Reserve Phenotype

**Figure 1:**
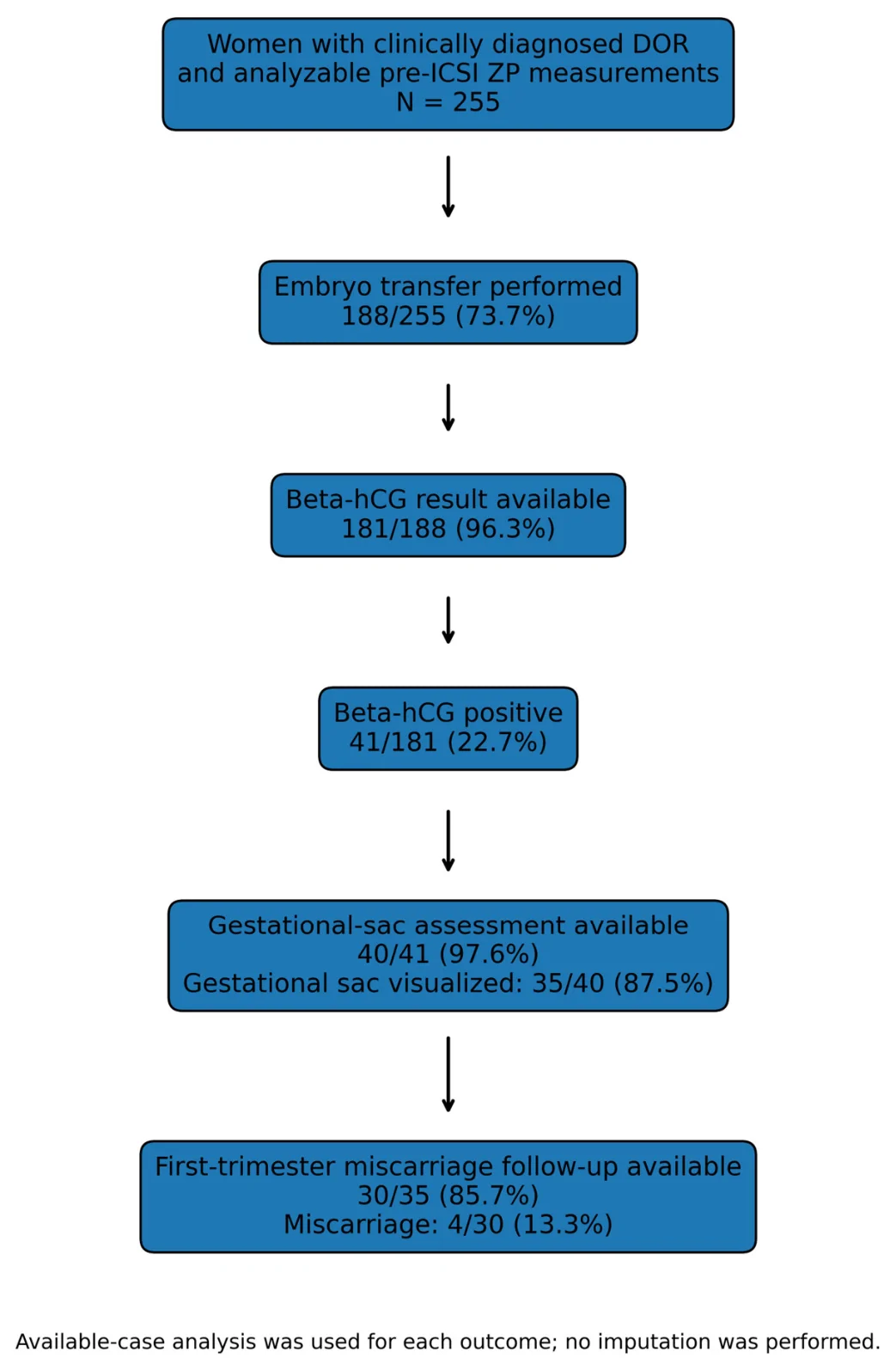
Study flow and available-case follow-up denominators. The figure shows the sequential availability of embryo transfer, beta-hCG, gestational sac assessment, and first-trimester miscarriage follow-up. No missing outcome values were imputed. DOR, diminished ovarian reserve; ICSI, intracytoplasmic sperm injection; ZP, zona pellucida; hCG, human chorionic gonadotropin.

The final analytic cohort included 255 women aged 22–46 years, comprising 117 women aged <35 years and 138 aged ≥35 years. Mean age was 35.5 ± 5.4 years. The ovarian reserve phenotype was quantitatively low: median AMH was 0.55 ng/mL (interquartile range, 0.31–0.82; range, 0.01–1.47), and 252/255 women (98.8%) had AMH < 1.1 ng/mL. Median AFC was 5 (interquartile range, 3–7) among 250 women with available AFC. Baseline and ovarian response characteristics are summarized in Table 1. The available-case follow-up sequence is shown in Figure 1.

### 3.2. Zona Pellucida Thickness and Reproductive Age

**Figure 2:**
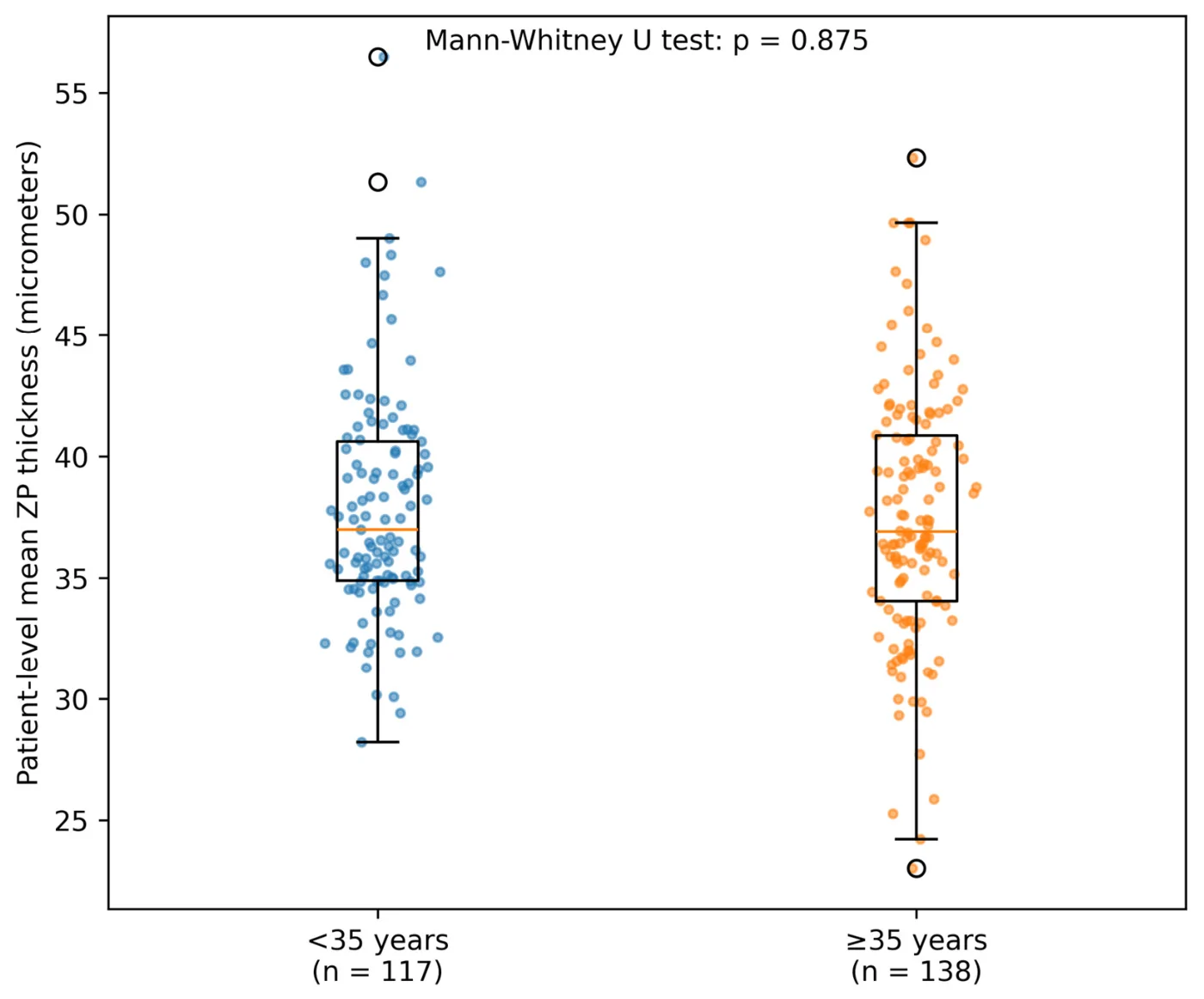
Distribution of patient-level mean pre-ICSI zona pellucida thickness according to reproductive age group. Boxes represent the median and interquartile range, whiskers extend to 1.5 times the interquartile range, and points represent individual participants. The between-group comparison used the Mann–Whitney U test. ICSI, intracytoplasmic sperm injection; ZP, zona pellucida.

Patient-level mean ZP thickness did not differ between age groups (36.99 [34.90–40.61] micrometers in women aged <35 years vs. 36.91 [34.04–40.87] micrometers in women aged ≥35 years; *p* = 0.875). Chronological age was also not meaningfully correlated with mean ZP thickness (Spearman rho = −0.059; *p* = 0.347). The distribution of patient-level mean ZP thickness across age groups is shown in Figure 2.

### 3.3. Ovarian Response, Transfer Context, and Early Reproductive Outcomes

Women aged <35 years had higher AMH levels, AFC, retrieved oocyte counts, and MII oocyte counts than women aged ≥35 years. Although the median number of 2PN embryos was identical in both groups, the distributions differed significantly (median [IQR]: 2 [1,2,3,4] vs. 2 [1,2]; *p* < 0.001). Maturation and fertilization rates were comparable between the groups (Table 1). Embryo transfer was performed in 91/117 (77.8%) younger and 97/138 (70.3%) older women (*p* = 0.176). Transfer day ranged from day 2 to day 5; the overall transfer day distribution did not differ significantly between age groups (*p* = 0.146). LAH was substantially more frequent in the older group (66/95, 69.5%) than in the younger group (32/90, 35.6%; *p* < 0.001).

Beta-hCG positivity was more frequent in women aged <35 years (27/85, 31.8%) than in women aged ≥35 years (14/96, 14.6%; *p* = 0.006). Among beta-hCG-positive pregnancies with recorded ultrasound follow-up, gestational sac visualization occurred in 24/26 (92.3%) younger and 11/14 (78.6%) older women. First-trimester miscarriage was recorded in 2/21 (9.5%) and 2/9 (22.2%), respectively (Table 2).

### 3.4. Zona Pellucida Thickness and Early Reproductive Outcomes

Mean ZP thickness was similar in participants with positive and negative beta-hCG results (36.73 [34.82–39.65] vs. 37.36 [34.85–40.96] micrometers; *p* = 0.953). ZP thickness also did not differ according to gestational sac visualization or first-trimester miscarriage (Table 3). Correlations between mean ZP thickness and AMH, MII oocyte count, and fertilization rate were weak and nonsignificant.

### 3.5. Transfer-Adjusted Multivariable Analysis

The multivariable model included 177 women with complete beta-hCG, LAH, transfer timing, MII oocyte, and ZP data. Patient-level mean ZP thickness was not independently associated with beta-hCG positivity (OR 1.03 per micrometer, 95% CI 0.95–1.11; *p* = 0.487). LAH and day-5 transfer were also not statistically significant. Age ≥ 35 years remained associated with lower odds of beta-hCG positivity (OR 0.39, 95% CI 0.17–0.88; *p* = 0.023) (Table 4).

In the sensitivity model substituting AMH for MII oocyte count, the ZP estimate remained materially unchanged (OR 1.02 per micrometer, 95% CI 0.95–1.10; *p* = 0.572). Thus, the absence of an independent ZP association was consistent across alternative parsimonious specifications, although the confidence intervals do not exclude small effects.

## 4. Discussion

### 4.1. Principal Biological Interpretation

This prospective study evaluated whether patient-level mean pre-ICSI ZP thickness was associated with reproductive age or early developmental competence in women with DOR. In this cohort, mean ZP thickness was similar between women aged <35 years and those aged ≥35 years and showed no significant association with chronological age when age was analyzed as a continuous variable. It was also not independently associated with beta-hCG positivity after adjustment for reproductive age, LAH, transfer timing, and MII oocyte count. These findings apply specifically to average pre-ICSI ZP thickness and do not exclude age-related differences in other structural, mechanical, or dynamic characteristics of the ZP. In contrast, reproductive aging was reflected more clearly in ovarian response and early pregnancy outcome, with lower AMH, AFC, oocyte yield, MII oocyte count, and beta-hCG positivity among older women.

The distinction is biologically important. Ovarian reserve markers principally describe follicular quantity and expected response, whereas age captures broader changes in oocyte competence [2,3,4]. Our findings suggest that the age-related deterioration observed in this DOR cohort is not mirrored by a simple monotonic change in average ZP thickness. Accordingly, mean thickness should not be treated as a structural surrogate for the wider oocyte-aging phenotype.

### 4.2. Mean Thickness Is Not Equivalent to Zona Pellucida Biology

The ZP is a three-dimensional glycoprotein matrix whose behavior depends on protein composition, fibrillar organization, cross-linking, porosity, elasticity, and dynamic remodeling [8,9,10,11,12,13,14,15,22]. A patient-level mean thickness value collapses this complexity into a single scalar measure. It cannot identify regional asymmetry, local weakening, changes in optical density, matrix stiffness, or the spatial pattern of thinning that precedes hatching. Therefore, a negative result for average thickness should not be interpreted as evidence that ZP biology is irrelevant to oocyte or embryo competence.

This interpretation helps reconcile our data with earlier studies. Nawroth et al. evaluated metaphase II oocytes and proposed an age-related contribution of a thicker ZP to lower fertilization in conventional IVF, whereas Balakier et al. found that embryo ZP thickness was not strongly influenced by female age or hormonal levels [16,21]. Sequential and variation-based analyses by Garside, Gabrielsen, and Sun emphasized dynamic or regional thickness behavior rather than the simple mean [17,18,19,20]. Our findings therefore narrow the question: in a clinically low-reserve ICSI cohort, patient-level mean pre-ICSI thickness was not a useful measurable phenotype of reproductive aging.

### 4.3. Reproductive Aging and Oocyte Competence in Diminished Ovarian Reserve

DOR is especially informative because quantitative scarcity magnifies the clinical importance of each oocyte, but it also complicates attempts to infer quality from visible morphology. In our cohort, almost all women had very low AMH values, yet age still separated ovarian response and early pregnancy outcome. This pattern is consistent with the concept that AMH and AFC are stronger markers of expected oocyte yield than of age-related competence [2,3,4]. The absence of an age–ZP thickness gradient further suggests that structural aging of the oocyte cannot be reduced to progressive thickening or thinning of the zona.

The cohort was clinically diagnosed with DOR rather than defined by Bologna or POSEIDON criteria [33,34]. This is not a semantic distinction. Bologna criteria were developed to standardize poor ovarian response, whereas POSEIDON stratifies low-prognosis patients using age, ovarian reserve, and ovarian response. Our study instead asked a phenotype question within a clinically low-reserve population. The descriptive AMH distribution confirms a markedly low-reserve cohort, but the lack of a prespecified single enrollment cutoff limits direct comparison with strictly classified populations.

### 4.4. Why the ICSI Context Matters

The clinical meaning of ZP thickness may differ between conventional IVF and ICSI. In conventional IVF, sperm binding, the acrosome reaction, and penetration through the ZP are required for fertilization [8,9,10,11]. ICSI bypasses these steps by directly injecting a spermatozoon into the oocyte. Therefore, a ZP characteristic that affects sperm–zona interaction may have less influence on fertilization after ICSI. In our study, fertilization rates were similar between the age groups, and mean ZP thickness was not significantly correlated with fertilization rate. These findings suggest that the relevance of ZP thickness may depend partly on the assisted reproduction procedure used.

At the same time, the ZP remains relevant after ICSI because it protects the early embryo and participates in hatching. This is precisely why static pre-ICSI thickness may be too distal from implantation biology. Dynamic thinning, biochemical hardening, matrix elasticity, and blastocyst expansion may be more directly related to post-fertilization competence than the starting average thickness measured at the MII oocyte stage.

### 4.5. Transfer Context, Assisted Hatching, and Early Outcome

Because LAH use differed substantially between age groups and transfer timing may influence post-transfer outcomes, both factors were included in the multivariable model. AMH and MII oocyte count were not entered simultaneously. The ZP estimate remained close to the null after adjustment. A sensitivity model using AMH instead of MII oocyte count produced the same interpretation. These analyses strengthen the conclusion that average ZP thickness is not a robust independent correlate of beta-hCG positivity in this dataset.

The higher use of LAH in older women most likely reflects confounding by indication and poorer baseline prognosis rather than a causal adverse effect of the procedure. Current systematic reviews and randomized evidence do not support routine assisted hatching for all IVF/ICSI patients [30,31,32]. Our transfer-adjusted results also argue against a simplistic biological model in which older oocytes have a thicker zona that necessarily requires manipulation. However, the absence of a validated fresh-versus-frozen transfer variable remains an important limitation, and the current study should not be interpreted as a treatment-effect analysis of LAH.

### 4.6. Strengths and Limitations

The strengths of this study include its prospective design, focused DOR population, standardized pre-ICSI measurement at three equidistant ZP positions, independent two-observer verification with protocolized remeasurement when disagreement exceeded 5%, and patient-level analysis that avoids pseudoreplication of multiple oocytes from the same woman. Transfer day and LAH were explicitly incorporated into the adjusted analysis, and model complexity was restricted in view of the number of beta-hCG-positive events.

Several limitations remain. First, this was a single-center observational study without a normal ovarian reserve control group. Second, DOR was diagnosed clinically using AMH and AFC rather than a single prespecified enrollment cutoff; the findings therefore apply to a clinically characterized low-reserve cohort and cannot be directly generalized to every Bologna or POSEIDON subgroup. Third, no a priori sample size calculation was documented. The achieved cohort could detect a small-to-moderate standardized age group difference, but the 41 beta-hCG-positive events limited multivariable precision, and small independent ZP effects cannot be excluded.

Fourth, the source dataset did not reliably distinguish fresh from frozen-thawed embryo transfer, and detailed embryo morphology or morphokinetic variables were not available for modeling. Transfer policies may therefore have contributed residual confounding. Fifth, follow-up denominators declined across beta-hCG, ultrasound, and miscarriage assessment. The exact follow-up sequence is reported, and available-case analysis was used without imputation; however, reasons for missing follow-up were not systematically coded, and selection bias remains possible. Sixth, although ZP measurements were independently verified by two observers, observer-specific raw values were not retained, preventing calculation of an intraclass correlation coefficient. Although chronological age was also analyzed as a continuous variable, the study was not designed to evaluate nonlinear age-related patterns or differences across narrow one- or two-year age intervals. Therefore, subtle or non-monotonic associations between age and ZP thickness cannot be excluded. Finally, the study evaluated average thickness rather than ZP thickness variation, density, birefringence, elasticity, or standardized qualitative abnormalities.

### 4.7. Future Directions

Future studies should move from one-dimensional thickness measurement toward multidimensional ZP phenotyping. Quantitative image analysis could integrate mean thickness, regional variation, symmetry, optical density, perivitelline-space morphology, and texture. These features should be linked with raw oocyte-level data, observer reliability, embryo grading, morphokinetics, transfer-cycle type, and live birth. Repeated measurements from the oocyte through blastocyst expansion may be especially informative because they would characterize ZP remodeling rather than a single static state.

From a reproductive-biology perspective, the most relevant next question is not whether the zona is simply thicker or thinner with age, but whether reproductive aging alters a composite matrix phenotype involving structure, mechanics, and dynamics. Such an approach may reveal biologically meaningful ZP signatures even when average pre-ICSI thickness alone is uninformative.

## 5. Conclusions

In this cohort of women with clinically diminished ovarian reserve undergoing ICSI, patient-level mean pre-ICSI ZP thickness was similar between women aged <35 years and those aged ≥35 years and was not significantly associated with chronological age when age was analyzed as a continuous variable. Mean ZP thickness was also not independently associated with beta-hCG positivity after adjustment for LAH, transfer timing, and ovarian response. These findings apply specifically to average pre-ICSI ZP thickness and do not exclude age-related changes in other structural, mechanical, or dynamic properties of the ZP. Reproductive aging was more clearly reflected in ovarian reserve, ovarian response, and early pregnancy outcome.

## Figures and Tables

**Table 1:** Baseline and ovarian response characteristics according to reproductive age group.

Variable	<35 Years (*n* = 117)	≥35 Years (*n* = 138)	*p* Value
Age, years	31.0 (28.0–33.0)	39.0 (38.0–40.8)	<0.001
Body mass index, kg/m^2^	23.7 (22.0–27.3)	24.6 (22.7–27.7)	0.082
Infertility duration, years	2.0 (2.0–3.0)	3.0 (2.0–4.8)	<0.001
FSH, IU/L	8.2 (6.7–10.6)	8.0 (6.3–10.7)	0.636
AMH, ng/mL	0.64 (0.47–0.90)	0.42 (0.22–0.79)	<0.001
Antral follicle count	7.0 (4.0–9.0)	4.0 (2.0–6.0)	<0.001
Retrieved oocytes, n	6.0 (3.0–8.0)	4.0 (2.0–5.0)	<0.001
Metaphase II oocytes, n	4.0 (2.0–6.0)	2.0 (2.0–4.0)	<0.001
Oocyte maturation rate, %	80.0 (60.0–100.0)	75.5 (60.5–100.0)	0.863
Two-pronuclear embryos, n	2.0 (1.0–4.0)	2.0 (1.0–2.0)	<0.001
Fertilization rate, %	66.0 (50.0–100.0)	66.0 (50.0–100.0)	0.967
Patient-level mean ZP thickness, micrometers	36.99 (34.90–40.61)	36.91 (34.04–40.87)	0.875
Measured oocytes contributing to ZP mean, n	4.0 (2.0–6.0)	2.0 (2.0–4.0)	<0.001

Values are median (interquartile range) unless otherwise indicated. *p* values were calculated using the Mann–Whitney U test. BMI was calculated after correction of a height-entry error; one participant remained excluded from BMI analyses because height and weight were missing. FSH, follicle-stimulating hormone; AMH, anti-Müllerian hormone; ZP, zona pellucida. For the 2PN embryo count, the identical group medians were accompanied by different distributions and interquartile ranges; the *p* value was derived from comparison of the full distributions using the Mann–Whitney U test.

**Table 2:** Clinical, transfer, and early reproductive outcomes according to reproductive age group.

Outcome	<35 Years	≥35 Years	*p* Value
Primary infertility	104/117 (88.9)	110/138 (79.7)	0.047
Secondary infertility	13/117 (11.1)	28/138 (20.3)	0.047
Previous infertility-related abortion	6/117 (5.1)	5/134 (3.7)	0.759
Embryo transfer performed	91/117 (77.8)	97/138 (70.3)	0.176
Transfer day 2	13/91 (14.3)	19/97 (19.6)	0.146 *
Transfer day 3	48/91 (52.7)	52/97 (53.6)	
Transfer day 4	14/91 (15.4)	19/97 (19.6)	
Transfer day 5	16/91 (17.6)	7/97 (7.2)	
Laser-assisted hatching performed	32/90 (35.6)	66/95 (69.5)	<0.001
Positive beta-hCG	27/85 (31.8)	14/96 (14.6)	0.006
Gestational sac visualized	24/26 (92.3)	11/14 (78.6)	0.322
First-trimester miscarriage	2/21 (9.5)	2/9 (22.2)	0.563

Values are n/N (%). The previous infertility-related abortion variable was available for 134 women in the ≥35-year group; other denominators vary according to transfer status and available post-transfer follow-up. * The *p* value refers to the overall four-category transfer day distribution. Categorical comparisons used the chi-square or Fisher’s exact test, as appropriate. hCG, human chorionic gonadotropin.

**Table 3:** Patient-level mean zona pellucida thickness according to early reproductive outcomes.

Outcome Category	n	Mean ZP Thickness, Micrometers	*p* Value
Positive beta-hCG	41	36.73 (34.82–39.65)	0.953
Negative beta-hCG	140	37.36 (34.85–40.96)	Reference
Gestational sac visualized	35	37.41 (34.85–39.95)	0.261
No gestational sac visualized	5	36.73 (33.26–37.75)	Reference
First-trimester miscarriage	4	38.07 (37.23–38.94)	0.659
No first-trimester miscarriage	26	36.67 (34.83–40.52)	Reference

Values are median (interquartile range). *p* values were calculated using the Mann–Whitney U test. ZP, zona pellucida; hCG, human chorionic gonadotropin.

**Table 4:** Transfer-adjusted multivariable logistic regression for beta-hCG positivity.

Predictor	Odds Ratio	95% Confidence Interval	*p* Value
Age ≥ 35 years	0.385	0.169–0.879	0.023
Patient-level mean ZP thickness, per micrometer	1.027	0.953–1.107	0.487
Laser-assisted hatching	1.458	0.648–3.280	0.362
Day-5 transfer vs. days 2–4	1.913	0.667–5.483	0.228
Metaphase II oocytes, per oocyte	1.105	0.954–1.280	0.184

The model included 177 women with complete covariate and beta-hCG data and 41 beta-hCG-positive events. AMH and MII oocyte count were not entered simultaneously. A sensitivity model substituting AMH for MII oocyte count yielded a similar ZP estimate (OR 1.021, 95% CI 0.949–1.099; *p* = 0.572). ZP, zona pellucida; hCG, human chorionic gonadotropin; AMH, anti-Müllerian hormone; MII, metaphase II.

## Data Availability

The datasets generated and/or analyzed during the current study are not publicly available because of privacy and ethical restrictions but are available from the corresponding author on reasonable request, subject to institutional approval and applicable data protection regulations.
